# Clinical Characteristics, Diagnostic Evaluation, and Antibiotic Prescribing Patterns for Skin Infections in Nursing Homes

**DOI:** 10.3389/fmed.2016.00030

**Published:** 2016-07-21

**Authors:** Norihiro Yogo, Gregory Gahm, Bryan C. Knepper, William J. Burman, Philip S. Mehler, Timothy C. Jenkins

**Affiliations:** ^1^Department of Medicine, Division of Infectious Diseases, University of Colorado, Aurora, CO, USA; ^2^University of Colorado School of Medicine, Aurora, CO, USA; ^3^Department of Medicine, Division of Geriatrics, University of Colorado, Aurora, CO, USA; ^4^Department of Patient Safety and Quality, Denver Health, Denver, CO, USA; ^5^Denver Health, Denver, CO, USA; ^6^Denver Public Health, Denver Health, Denver, CO, USA

**Keywords:** antimicrobial stewardship, nursing homes, long-term care facilities, skin and soft tissue infection, skin infections

## Abstract

**Background:**

The epidemiology and management of skin infections in nursing homes has not been adequately described. We reviewed the characteristics, diagnosis, and treatment of skin infections among residents of nursing homes to identify opportunities to improve antibiotic use.

**Methods:**

This was a retrospective study involving 12 nursing homes in the Denver metropolitan area. For residents at participating nursing homes diagnosed with a skin infection between July 1, 2013 and June 30, 2014, clinical and demographic information was collected through manual chart review.

**Results:**

Of 100 cases included in the study, the most common infections were non-purulent cellulitis (*n* = 55), wound infection (*n* = 27), infected ulcer (*n* = 8), and cutaneous abscess (*n* = 7). In 26 cases, previously published minimum clinical criteria for initiating antibiotics (Loeb criteria) were not met. Most antibiotics (*n* = 52) were initiated as a telephone order following a call from a nurse, and 41 patients were not evaluated by a provider within 48 h after initiation of antibiotics. Nearly all patients (*n* = 95) were treated with oral antibiotics alone. The median treatment duration was 7 days (interquartile range 7–10); 43 patients received treatment courses of ≥10 days.

**Conclusion:**

Most newly diagnosed skin infections in nursing homes were non-purulent infections treated with oral antibiotics. Antibiotics were initiated by telephone in over half of cases, and lack of a clinical evaluation within 48 h after starting antibiotics was common. Improved diagnosis through more timely clinical evaluations and decreasing length of therapy are important opportunities for antibiotic stewardship in nursing homes.

## Introduction

Antibiotic resistance and *Clostridium difficile* infection have been identified as urgent health threats by the Centers for Disease Control and Prevention (1) and have resulted in the need to develop systems to improve antibiotic use as a national priority (2, 3). The Infectious Diseases Society of America/Society for Hospital Epidemiology of America (IDSA/SHEA) provide broad recommendations for antimicrobial stewardship interventions (2). These guidelines highlight the importance of improving antibiotic use not only in acute care hospitals but also in nursing homes and long-term care facilities (2, 3). Recent nationwide surveys in the United States have demonstrated that 12% of nursing home residents have an infection at any given time (4), and up to 79% of nursing home residents receive an antibiotic over the course of a year (5). An estimated 25–75% of antibiotic use in such facilities is deemed to be inappropriate (6, 7). Antibiotic exposure in long-term care facilities is associated with the acquisition of drug-resistant organisms (8, 9). Furthermore, there is a markedly increased risk of *C. difficile* infection in the elderly, and an estimated one-quarter of all cases in the United States occur in nursing homes (10). Given that 1.4 million individuals are currently residing in 15,700 nursing homes in the Unites States (11), antimicrobial stewardship in nursing homes is essential to prevent these common complications of antibiotic use.

The most common indications for antibiotics in nursing homes include urinary tract, respiratory tract, and skin and soft tissue infections (4, 12). Thus far, studies to improve antibiotic use in nursing homes have focused largely on urinary tract infections (13–15) and pneumonia (13, 16, 17). In both hospitalized patients and outpatients, skin and soft tissue infections are commonly associated with inappropriate antibiotic use (18–20); however, studies on the diagnosis and management of skin infections in the nursing home setting are lacking. The objectives of this study were to describe the epidemiology, clinical characteristics, diagnostic evaluation, and antibiotic prescribing patterns for skin infections in nursing homes to identify opportunities to improve antibiotic utilization.

## Materials and Methods

This was a retrospective study of skin infections involving 12 nursing homes within the Denver metropolitan area between July 1, 2013 and June 30, 2014. The 12 participating facilities ranged in size from 60 to 160 patients, with a combined census of 985 patients [818 (83%) long-term residents]. The patients at these facilities were managed by 67 unique providers: 36 physicians and 31 nurse practitioners or physician assistants. On-site wound care consultation was available at all participating facilities at the discretion of the patient’s provider. None of the nursing homes were involved in any other antimicrobial stewardship initiatives either prior to or during the study period. This study was approved by the Colorado Multiple Institutional Review Board.

### Case Identification and Data Collection

Residents identified as having any skin infection in sections I or M of the Minimum Data Set (MDS) (per the facility’s usual documentation practices) and initiated on systemic antibiotic therapy were identified for review. Cases involving continuation of therapy from a hospitalization or outside facility, osteoarticular infections, necrotizing soft tissue infections, use of prophylactic or suppressive antibiotic therapy, and existence of a concurrent infection requiring antibiotic therapy were excluded. Cases with unavailable or incomplete medical records were also excluded. For patients who had multiple episodes of a skin infection during the study period, only the initial treatment course was reviewed.

For cases meeting study entry criteria, demographic information, selected comorbid conditions, diagnostic data, antibiotic therapy, and clinical outcomes during a 30-day follow-up period from the start of treatment were manually recorded using a standardized documentation form. Clinical characteristics of the skin infections were determined through review of clinician and nursing documentation. The antibiotic selection and duration were collected through review of nursing home provider order forms and medication administration records.

### Cases Classifications and Definitions

Skin infections were categorized as non-purulent cellulitis, purulent cellulitis, cutaneous abscess, wound infection, or an infected ulcer as documented in provider and/or nursing notes. The Loeb minimum criteria for the initiation of antibiotics for skin infections was defined as new or increasing purulent drainage; or at least two of the clinical features of fever, redness, tenderness, warmth, or new or increased swelling of the affected site (21). Antibiotics were deemed to have been initiated through a call from a nurse if a call to a provider resulted in a verbal order for antibiotics. Antibiotics were considered to be initiated by a non-nursing home provider if they were ordered by a wound care physician, emergency room provider, or at an outside clinic visit. Documentation of an in-person evaluation within 48 h of the start of antibiotic therapy by any provider – physician, nurse practitioner, physician assistant, or non-nursing home provider – was also recorded. Antibiotics with activity against methicillin-resistant *Staphylococcus aureus* (MRSA) were defined as tetracyclines, clindamycin, trimethoprim-sulfamethoxazole, vancomycin, linezolid, and daptomycin. Antibiotics with a broad-spectrum of Gram-negative activity were defined as β-lactam/β-lactamase inhibitor combinations, second- or third-generation cephalosporins, carbapenems, and fluoroquinolones. Treatment failure was defined as a change in antibiotic regimen or extension of treatment duration due to inadequate clinical response. Relapse within the 30-day follow-up period was defined as recurrence of infection in the same anatomic location after completion of initial therapy. Any adverse events attributed to antibiotics, including *C. difficile* infection, were recorded.

### Data Analysis

The main outcomes of interest included the frequency of the various types of skin infections, the frequency with which antibiotics were started without a clinical evaluation, and antibiotic selection and duration of therapy. Descriptive statistics were calculated for the entire cohort. Given expected differences in the treatment of non-purulent cellulitis as compared with purulent infections (abscess, purulent cellulitis, wound infection, and infected ulcers), analyses were also stratified by these two groups. Odds ratios (OR) and 95% confidence intervals (CI) were used to assess the association with categorical variables. For continuous variables, the Wilcoxon rank-sum test was used. All analyses were performed using SAS version 9.3 (SAS Institute, Cary, NC, USA).

## Results

In total, 135 cases were reviewed. Thirty-five cases were excluded for the following reasons (more than one may have been present): continuation of therapy from an outside institution (*n* = 13), presence of a concurrent infection requiring antibacterial therapy (*n* = 12), confirmed or suspected osteomyelitis or necrotizing infection (*n* = 5), incomplete medical records (*n* = 4), suppressive or prophylactic antibiotic use (*n* = 3), multiple skin infections during the study period (*n* = 1). The remaining 100 cases were included in the final study population. Demographic and clinical characteristics are shown in Table 1. The most common comorbidities included lymphatic or vascular disease, dementia, and diabetes mellitus.

**Table 1:** **Baseline demographic and clinical characteristics**.

	**Total cases** ***n*** **= 100**
Age (mean, 95% confidence interval)	72.2 (69.4–75.0)
Male	38
Dementia	41
Diabetes mellitus	39
Lymphatic or vascular disease^a^	55
Wheelchair-dependence	54
Bedridden	9
Hospitalization within 30 days	37
Skin infection within 30 days	9
History of MRSA infection and/or colonization	8

*^a^Defined as chronic venous stasis, lymphedema, peripheral arterial disease, or chronic lower extremity edema*.

The most common type of skin infection was non-purulent cellulitis (*n* = 55, Table 2); in 8 of these cases, the diagnosis was “bilateral lower extremity cellulitis.” Wound infection was the next most common (*n* = 27), of which 19 were postoperative. There were eight cases of infected ulcer, seven cases of cutaneous abscess, and one case of purulent cellulitis. Anatomically, most cases involved the extremities (*n* = 81), 69 of which involved the lower extremities. The Loeb minimum criteria for the initiation of antibiotics were not met in 26 patients. Thirty patients had a white blood cell count performed, and 12 had a level ≥11,000 cells/mm^3^. Cultures were obtained in only 10 cases, of which 5 yielded a clinically significant isolate; therefore, antibiotic therapy in the vast majority of cases was empiric.

**Table 2:** **Type and clinical features of skin infections**.

	**Total cases** ***n*** **= 100**
**Type of skin infection^a^**	
Non-purulent cellulitis	55
Wound infection	27
Infected ulcer	8
Cutaneous abscess	7
Purulent cellulitis	1
Other skin infection	7
Recurrence of a prior skin infection	7
**Initial location of involvement^b^**	
Extremities	81
Leg	58
Foot or ankle	11
Hand or wrist	9
Arm	4
Trunk	15
Abdomen	5
Buttock/hip	6
Back	4
Chest	2
Head and neck	4
Groin/inguinal	2
**Types of wounds associated with skin infection**	
Postoperative wound	19
Skin tear or abrasion	12
Ulcer	9
Other	18
**Clinical features**	
Temperature ≥100.0°F	3
Erythema	81
Induration	31
Warmth	43
Pain	47
Gross purulence	13
Absence of Loeb minimum criteria^c^	26

*^a^Includes five cases with two different types of concurrent skin infection*.

*^b^Includes five cases with skin infection in more than one anatomic site*.

*^c^New or increasing purulent drainage; or at least two of the clinical features of fever, redness, tenderness, warmth, or new or increased swelling of the affected site (21)*.

Most antibiotics (*n* = 52) were initiated as a telephone order following a call from a nurse; 41 patients were not evaluated by any provider within 48 h after initiation of antibiotics. Sixteen patients had their antibiotics initiated by a non-nursing home provider. Fifteen patients had a wound care consultation, and only one patient had an infectious diseases specialist consultation to assist in management. Ninety-five patients were treated with oral antibiotics only, and five patients received treatment that involved intravenous antibiotics (Table 3). The most common class of antibiotics prescribed was an oral β-lactam (*n* = 73); 28 cases were prescribed an oral antibiotic with activity against MRSA; and 18 patients received antibiotics with broad Gram-negative activity.

**Table 3:** **Antibiotic selection, duration of therapy, and clinical outcomes**.

	**Total cases** ***n*** **= 100**
**Antibiotic selection^a^**	
Oral β-lactam	73
Cephalexin	65
Amoxicillin/clavulanate	5
Other oral β-lactam	4
Oral anti-MRSA antibiotic	28
Doxycycline or tetracycline^b^	17
Trimethroprim-sulfamethoxazole	7
Clindamycin	5
Fluoroquinolone^c^	10
Intravenous antibiotic	5
Vancomycin	2
Ertapenem	2
Ceftriaxone or cefazolin	2
Antibiotic with broad Gram-negative activity^d^	18
Multiple antibiotics prescribed	18
Sequential therapy	12
Combination therapy	6
**Treatment duration**	
Total duration of therapy, median days (IQR)	7 (7–10)
Duration of therapy ≥10 days	43
Duration of therapy ≤5 days	6
**Clinical outcomes**	
Treatment failure	15
Required hospitalization	4
Discharged to home with antibiotics	3

*^a^Totals for subgroups include 18 patients who received more than 1 antibiotic*.

*^b^Includes doxycycline use in 16 cases and tetracycline in 1 case*.

*^c^Includes levofloxacin use in nine cases and moxifloxacin in one case*.

*^d^Defined as β-lactam/β-lactamase inhibitor combinations, second- or third-generation cephalosporins, carbapenems, and fluoroquinolones*.

When comparing the management of non-purulent cellulitis with other infection types, antibiotics with activity against MRSA were used with similar frequency [24 vs. 33%, respectively; OR 0.62 (95% CI: 0.26–1.49)]; however, patients with non-purulent cellulitis were less likely to be prescribed antibiotics with broad Gram-negative activity [9 vs. 31%; OR 0.22 (95% CI: 0.07–0.68)]. Overall, the median duration of therapy was 7 days [interquartile range (IQR) 7–10]. In 43 cases, the prescribed duration was ≥10 days. Treatment failure occurred in 15 cases, and 4 patients required admission to an acute care hospital. Relapse within the 30-day follow-up period occurred in 11 cases. An adverse drug event was documented in 11 cases including gastrointestinal side effects in 10 and a rash in one. No patient was diagnosed with *C. difficile* infection during the follow-up period.

## Discussion

Understanding the clinical characteristics, diagnostic evaluation, and treatment of skin infections in nursing homes is essential to optimize antibiotic use for these common conditions. A number of prior studies have evaluated overall antibiotic prescribing practices in nursing homes and long-term care facilities (22–26); however, to our knowledge, this is the first detailed description of the treatment of skin infections in this population.

The most common type of skin infection in this study was non-purulent cellulitis, accounting for over half of all cases. This contrasts sharply to hospitals and ambulatory care, where a substantially higher proportion of purulent infections has been observed (19, 20). Furthermore, in this study, evidence of systemic involvement was uncommon; almost all cases were treated with oral antibiotics alone, and only four cases required hospitalization. These findings suggest that the majority of skin infections in this population were mild, uncomplicated infections.

The diagnosis of skin infections can be challenging. In this study, the most common anatomical location of infection was the lower extremities, a finding that is not surprising since over half of patients had lymphatic or vascular disease and over half were wheelchair dependent. Such vascular insufficiency is frequently associated with conditions that mimic skin infections (e.g., venous stasis dermatitis) and result in misdiagnosis of cellulitis (27). It is notable that eight patients were diagnosed as having “bilateral lower extremity cellulitis,” an uncommon clinical phenomenon, which suggests that the diagnosis of cellulitis was inaccurate in some patients. Due to the difficulty of diagnosing infections in the elderly, many of whom may have atypical signs and symptoms of an infection, a set of minimum criteria for the initiation of antibiotics for suspected infection (Loeb criteria) have previously been developed (21). Similar to a previous study, we found that these minimum criteria for skin infection were often not met (26 cases in our study), despite the initiation of antibiotic therapy (22). In aggregate, our findings suggest that overdiagnosis of skin infections is likely common and highlight the need for interventions to improve diagnostic accuracy in order to avoid unnecessary antibiotic therapy for conditions that mimic skin infections.

One factor that may have contributed to the apparent overdiagnosis of skin infections in these nursing homes is the lack of timely clinical evaluations, either at the time of or shortly after the initiation of antibiotics. We demonstrated that over half of antibiotic courses were initiated as telephone orders, a finding similar to previous studies, where 54–67% of antibiotics were initiated over the telephone (23, 24). Moreover, nearly half of all patients were not evaluated by a provider within 48 h of the initiation of antibiotics. This is consistent with prior studies that showed only 44% of nursing home residents had a physician visit within 1 day of starting antimicrobial therapy (25), 44% had no documentation of an infection (24), and 56% did not have a documented physical examination (26). Clearly, in order to improve antibiotic use in nursing homes, processes that promote timely clinical evaluations to increase the accuracy of initial diagnosis and allow monitoring of response to antibiotic therapy are essential.

Overall, 95% of patients were treated in the absence of microbiological data to guide antibiotic therapy. This is likely due to the fact that most cases were non-purulent cellulitis, which is most often not able to be cultured; in contrast, purulent infections, such as abscesses, were relatively uncommon. Consistent with national guideline recommendations for the treatment of non-purulent cellulitis (28), the most commonly used antibiotics in this study were oral β-lactams. The use of antibiotics with activity against MRSA was less common (<30% of cases), and notably, there was no difference in use between non-purulent cellulitis and purulent infections where MRSA is common, such as abscesses, purulent cellulitis, wound infections, and infected ulcers. This suggests that the risk for MRSA was not a determinant in choosing an antibiotic regimen, and thus, antibiotic use could be improved through promoting appropriate use of MRSA-active agents in purulent infections. Likewise, 18 patients received broad-spectrum Gram-negative therapy, and 18 were treated with multiple antibiotics. Since national guidelines recommend the use of a single agent targeted toward Gram-positive pathogens (28), there may be an opportunity to reduce use of overly broad-spectrum antibiotic regimens in nursing home residents with skin infections. Although the median duration of antibiotic therapy was 7 days, over 40% of patients were treated for 10 days or longer. This is of importance since a treatment course of 5 days is currently recommended for uncomplicated skin infections (28); therefore, shortening treatment durations represent another important opportunity to reduce antibiotic exposure.

Nursing homes provide unique challenges and barriers to optimize antibiotic use, such as clinical providers being located off-site (6). This highlights the need for interventions to be specifically tailored for the nursing home setting to overcome these obstacles in order to be successful (2, 6). Effective interventions studied to date have included the implementation of practice guidelines (13, 14, 16, 17), clinician education (29), regular infectious diseases specialist consultation (30), and prospective audit with prescribing recommendations *via* telemedicine (31). It is notable that each of these interventions could potentially be applied – alone or in combination – to improve the management of skin and soft tissue infections in nursing homes. This type of infection-specific approach to antibiotic stewardship has been advocated by the CDC (32). Given the findings of this study, we believe that future nursing home interventions to reduce antibiotic overuse for skin and soft tissue infections need to incorporate early clinical evaluations to be most effective.

Our study has several important limitations. First, we retrospectively identified cases at each facility using the MDS, and as such, this study may not have captured every skin infection that occurred over the study period. Indeed, the limited number of included cases in this study suggests that the use of MDS was not likely a comprehensive surveillance method for new skin infections. Second, the retrospective study design with reliance on medical record documentation may have led to misclassification of cases or incomplete information. Likewise, applying the Loeb minimum criteria for this study was subject to the accuracy in assessment and documentation of the nursing home staff. Third, the study involved nursing homes in the Denver metropolitan area only; this may limit the generalizability of our findings, since local antimicrobial susceptibility patterns and antibiotic prescribing behaviors may differ elsewhere. Lastly, the small sample size did not allow for individual subgroup analysis for the different types of skin infections.

In summary, most skin infections in nursing homes appeared to be relatively mild cases of non-purulent cellulitis. Over half of all antibiotics were initiated over the telephone after a call from a nurse, nearly half of all cases had no clinical assessment by a provider within 48 h after initiation of antibiotics, and over one-quarter of cases did not meet the Loeb minimum criteria for the initiation of antibiotics. Antibiotic therapy was frequently broader in spectrum and more prolonged than recommended in national guidelines. Our findings suggest that skin infections represent an important opportunity for antibiotic stewardship in nursing homes and that prescribing could be improved through interventions that improve the accuracy of diagnosis, facilitate more timely clinical evaluations, and promote shorter courses of antibiotic therapy with Gram-positive activity.

## Author Contributions

NY is the lead investigator and headed study design, data collection, and primary authorship of the manuscript. GG is the medical director at each participating facility and participated in study design, facilitated access to medical records, and participated in manuscript review. BK is the primary statistician and helped with study design, statistical analysis, and participated in manuscript review. WB and PM are coinvestigators and participated in study design and manuscript review. TJ is the senior mentor of the study and oversaw study design, participated in statistical analysis, and is the primary editor of the manuscript.

## Conflict of Interest Statement

The authors declare that the research was conducted in the absence of any commercial or financial relationships that could be construed as a potential conflict of interest.
